# Pre-injury health status of injured patients: a prospective comparison with the Dutch population

**DOI:** 10.1007/s11136-018-2035-9

**Published:** 2018-10-30

**Authors:** Max W. de Graaf, Inge H. F. Reininga, Klaus W. Wendt, Erik Heineman, Mostafa El Moumni

**Affiliations:** 1 Department of Trauma Surgery, University Medical Center Groningen, University of Groningen, PO Box 30 001, 9700 RB Groningen, The Netherlands; 2 Department of Surgery, University Medical Center Groningen, University of Groningen, PO Box 30 001, 9700 RB Groningen, The Netherlands

**Keywords:** Short musculoskeletal function assessment, EQ-5D, Patient-reported outcome, Pre-injury, General population, The Netherlands, Trauma

## Abstract

**Purpose:**

The aim of this study was to assess whether injured patients have a different pre-injury health status compared to the Dutch population.

**Methods:**

A broad range of injured patients (age ≥ 18 and ≤ 75 years) completed the condition-specific Short Musculoskeletal Function Assessment (SMFA-NL) and generic health-related quality of life questionnaire EuroQol-5D (EQ-5D), within 2 weeks after patients sustained an injury. Patients reported their health status of the week before their injury. Scores were compared to the Dutch normative data of the questionnaires. Gender, age, educational level, relationship status, and comorbidity adjusted differences were calculated for the SMFA-NL.

**Results:**

A total of 596 injured patients completed the questionnaires (response rate: 43%). Unadjusted pre-injury SMFA-NL scores of injured patients were significantly better compared to the Dutch normative data (ranging from + 2.4 to + 8.6 points, *p* < 0.001 for all subscales and indices). The unadjusted EQ-5D difference score was 0.05 points (*p* < 0.001) higher in the group of injured patients. Adjusted pre-injury scores were higher than the SMFA-NL normative data. Function index: + 3.6, *p* < 0.001, bother index: + 3.0, *p* < 0.001 upper extremity dysfunction: + 0.8, *p* = 0.2, lower extremity dysfunction: + 3.7, *p* < 0.001. Problems with daily activities: + 2.8, *p* = 0.001. Mental and emotional problems: + 6.8, *p* < 0.001.

**Conclusions:**

Injured patients reported a better pre-injury health status compared to the Dutch population. Patient characteristics explained an important part of the difference in health status between injured patients and the Dutch population.

## Background

General health and physical functioning are frequently assessed in injured patients using patient-reported outcomes (PROMs) [1–6]. To clinicians, it is important to be able to evaluate to what extent patients have returned to their pre-injury health status. To assess changes in health status of injured patients, information about their pre- and post-injury health state values is needed. However, in acute-onset conditions such as acute traumatic injuries (as opposed to chronic conditions), data about pre-injury health status are usually not available. Though preferred, in day-to-day clinical practice, it is not feasible to prospectively collect data about pre-onset health status of patients that will become injured.

Although not a measurement property of a PROM (like validity and reliability), interpretability is a prerequisite for a proper use of a measurement instrument. Interpretability is the degree to which one can assign qualitative meaning (i.e., clinical or commonly understood connotations) to an instrument’s quantitative scores or change in scores [7]. To interpret the change in health status due to injury, different methods may be used. First, population-based normative data can be used as reference of pre-onset health status. Second, recalled pre-injury health state values reported shortly after sustaining the traumatic injury can be used as a proxy for pre-injury health status [8, 9]. Finally, health state values of a matched non-injured group of patients can be used to assess changes in health of injured patients [10].

Studies that compared recalled pre-injury health status to the general population using generic Health-related Quality of Life (HRQoL) questionnaires generally reported that recalled pre-injury health status was higher than the health status of the general population [8, 9, 11–13]. In these studies, it was suggested that injured patients may not be accurately reflected by population norms. However, it is not known if these findings may be generalized to more specific domains of health status, such as physical functioning, which is usually more affected in injured patients. Furthermore, previous studies compared pre-injury health status and normative data without adjustment of differences in general characteristics [8, 9, 11–13]. In other words, it is not known whether the reported differences remain after adjusting for the differences in general characteristics.

Two frequently used PROMs are the Short Musculoskeletal Function Assessment (SMFA) and the EQ-5D. The SMFA is a condition-specific questionnaire that was developed to assess physical functioning of patients with a variety of musculoskeletal disorders [14]. The EQ-5D is a generic HRQoL questionnaire that can be used to evaluate general health status.

The aims of this study were (1) to evaluate and report recalled pre-injury health status of injured patients using both the condition-specific SMFA and the generic HRQoL instrument EQ-5D, and (2) to investigate whether differences in health state values existed between injured patients and the Dutch population normative data.

## Materials and methods

### Patients

A prospective cohort study design was used. Injured patients were recruited at the emergency department of the University Medical Center Groningen (The Netherlands), a level 1 trauma center with an emergency department that is also open to self-referrals. Patients that presented with an acute injury due to trauma were prompted for inclusion. Patients had a broad range of acute injuries including wounds, fractures, or organ injury such as liver rupture or pneumothorax. Patients were identified as injured by a triage nurse of the emergency department and were treated by a surgery resident or trauma surgeon. Exclusion criteria were patients of age ≤ 17 or age > 75, inability to read and write Dutch, severe mental disabilities, traumatic brain injury with neurological symptoms, and patients that lived outside of The Netherlands. Eligible patients were requested to complete the SMFA-NL and EQ-5D questionnaires on paper within 2 weeks after the injury. Patients were asked to report their health status of the week before their injury. Non-responders were reminded once.

There are no clear guidelines regarding the required sample size for the comparison of normative data of PROMs to other samples. It has been recommended to use a sample size of at least 50 per age group to establish normative data [15]. The methods employed in this study have been reviewed by the local Institutional Review Board, and waived further need for approval (METc2012.104). The study was carried out in compliance with the principles outlined in the Declaration of Helsinki on ethical principles for medical research involving human subjects.

### Questionnaires

The original American SMFA consists of 46 items, divided into two indices: the *function index* (34 items) and the *bother index* (12 items) [14]. Reininga et al. cross-culturally adapted the SMFA into Dutch (SMFA-NL) and showed that it consists of four subscales: the *upper extremity dysfunction* (6 items), *lower extremity dysfunction* (12 items), *problems with daily activities* (20 items), and *mental and emotional problem* (8 items) subscales [16]. The original division into two indices is applicable for the Dutch SMFA-NL as well. Items were scored on a 1- to 5-point Likert scale. The SMFA-NL has been shown to be valid and reliable in injured patients [16]. In accordance with the SMFA-NL normative data, SMFA-NL scores were transposed to a 0 to 100 scale, with higher scores representing better function of patients in the explored domain.

The EQ-5D consists of 5 items (*mobility, self-care, daily activities, pain*, and *anxiety or depressive symptoms*) which are scored on a 1- to 3-point Likert scale [17, 18]. All five items load on one index value, calculated by the Dutch EQ-5D scoring algorithm [17]. Scores range from − 0.33 to 1.00, where 0.00 represents death and 1.00 represents the best possible health state. Scores below 0.00, representing a possible health state worse than death, are a consequence of the time trade-off method scoring algorithm [17, 19]. The EQ-5D has been demonstrated valid and reliable in injured patients and is available in Dutch [18, 20–22].

Patient-reported demographic characteristics are gender, age, relationship status, and educational level. Patients were asked to report the presence of 12 common chronic health conditions (migraine, hypertension, asthma or COPD, severe spinal conditions, severe gut-related diseases, osteoarthritis, rheumatoid arthritis, diabetes mellitus, stroke, myocardial infarction, severe non-infarct cardiac conditions, and malignant disease) as used in the health surveys of Statistics Netherlands [23, 24].

### Normative data

The SMFA-NL pre-injury scores were compared to the Dutch population normative data of the SMFA-NL [25]. The Dutch normative data of the SMFA-NL have been published in 2015 and were based on a population sample of 875 Dutch citizens. Participants were recruited per e-mail and completed the web-based questionnaire. The sample was considered an accurate reflection of the Dutch population based on the distribution of gender, age, educational level, relationship status, and prevalence of comorbidities. The dataset of the SMFA-NL population normative data was obtained and was used in the statistical analysis of this study. EQ-5D scores gathered in this study were compared to the Dutch normative data of the EQ-5D, published by Stolk et al. [26]. The EQ-5D normative data originate from 2009 and consisted of a sample of 2667 Dutch citizens. The majority of these normative data were sampled through a web-form. A small fraction of the data (*n* = 309) was obtained through an interview. The sample was considered an accurate reflection of the Dutch population. The original EQ-5D dataset could not be obtained; hence all analyses were performed using the data provided in the original publication [26].

### Data analysis

Demographic characteristics, injury type, and injury mechanism were presented as frequencies and proportions. The average number of chronic health conditions per patient was calculated. Means, standard deviations, and 95% confidence intervals were calculated for indices and subscales of both questionnaires. Six age groups were constructed (18–24, 25–34, 35–44, 45–54, 55–64, 65–75). The last age group did not continue the 10-year age band, matching the SMFA-NL normative data. EQ-5D normative data originally were stratified in 5-year age groups [26]. The mean and standard deviations of the EQ-5D scores of the normative data were pooled by weight of the number of participants in each 5-year age group to create the following age groups: 20–24, 25–34, 35–44, 45–54, 55–64, and 65–74 [27]. When 15% or more of the patients reported a maximal score on a subscale, a ceiling effect was considered to be present [28].

### Statistical analysis

For each subscale of the SMFA-NL and EQ-5D, the unadjusted difference in score between the injured patients and the Dutch population was compared using independent *t* tests. Multivariable linear regression analyses were used to evaluate the adjusted differences in the SMFA-NL subscale scores between the injured patients and the Dutch general population. The overall mean differences in scores between the injured patients and the Dutch population were adjusted for the covariables: gender, age, relationship status, educational level, and the number of chronic health conditions. The adjusted differences could not be calculated for the EQ-5D since the original dataset could not be obtained.

#### Sensitivity analysis

A two-part model approach was used to investigate the difference between injured patients and the Dutch population with respect to possible ceiling effects [29, 30]. In the first part of the two-part model, a multivariable logistic regression was used to estimate the (adjusted) difference in probability of achieving the maximum SMFA-NL score, between the injured patients and the Dutch population. The second part was a multivariable linear regression analysis to evaluate the differences in the SMFA-NL scores between the injured patients and the Dutch general population, among those with a sub-maximal SMFA-NL score (less than 100 points). In both parts of the two-part model, the covariables were gender, age, relationship status, educational level, and the number of chronic health conditions. The sensitivity analysis was performed for all indices and subscales of the SMFA-NL.

Missing values were handled listwise. Items that were answered incorrectly were handled as missing. A *p* value smaller than 0.05 was considered statistically significant. To correct for multiple comparisons in the multivariable regression analyses, a Bonferroni correction was used and the *p* value was set at 0.0083 (0.05/6).

## Results

### General characteristics

Between October 2012 and February 2014, a total of 596 patients filled in the questionnaires (response rate: 43%). All age groups contained at least 51 patients. Demographic characteristics, injury types, and injury mechanisms of the study sample are described in Table 1. The study sample contained more males (60%, *n* = 359) than females. Upper and lower extremity fractures were the most prevalent injuries (21% and 19%, respectively). Most patients sustained the injury in a traffic accident (22%), fall (22%), or during sports (21%). Of the injured patients, 54% reported that they did not have any chronic health condition (Table 1). The general characteristics of SMFA-NL and EQ-5D normative data sample are shown in Table 1 [25, 26].

**Table 1:** General characteristics

	Injured patients	SMFA-NL normative data [25]	EQ-5D normative data [26]
	*N* (%)	*N* (%)	*N* (%)
Gender			
Male	359 (60)	420 (50)	1331 (50)
Female	237 (40)	420 (50)	1336 (50)
Age groups			
18–24	133 (22)	146 (17)	217 (9)^a^
25–34	92 (15)	141 (16)	556 (23)
35–44	104 (17)	148 (17)	615 (26)
45–54	100 (17)	138 (16)	512 (21)
55–64	116 (20)	143 (17)	306 (13)
65–75	51 (9)	148 (17)	202 (8)^b^
Relationship status			
Single	217 (47)	253 (31)	
With partner	244 (53)	558 (69)	
Educational level			
Elementary school	31 (7)	22 (3)	
High school	172 (37)	307 (35)	
College	119 (26)	268 (31)	
Bachelor’s degree or higher	130 (28)	267 (31)	
Other	11 (2)		
Chronic health conditions			
None	237 (54)	321 (41)	
One	122 (28)	228 (29)	
Two	54 (12)	127 (16)	
Three or more	23 (5)	109 (14)	
Injuries			
Fracture			
Upper extremity	114 (21)		
Lower extremity	107 (19)		
Pelvis and sacrum	4 (1)		
Spine	16 (3)		
Other (incl. rib fractures)	7 (1)		
Sprain, luxation, and rupture	89 (16)		
Contusion	82 (15)		
Minor head and facial injuries	12 (2)		
Wounds	85 (15)		
Organ injury (incl. pneumothorax)	8 (1)		
Other	32 (6)		
Injury mechanism			
Traffic accidents			
Motorvehicle	57 (10)		
Bicycle	68 (12)		
Falls			
Work related	11 (2)		
At home	71 (13)		
Other	37 (7)		
Work related (non-fall)	46 (8)		
At home (non-fall)	64 (12)		
Sports injury	117 (21)		
Violence	23 (4)		
Other (non-fall)	61 (11)		

^a^In this age group, participants were aged 20–24

^b^In this age group, the participants were aged 65–74

### Difference in pre-injury health status injured patients and health status of the Dutch population

Unadjusted pre-injury scores of the injured patients were significantly better on all indices and subscales of the SMFA-NL, compared to the Dutch normative data. (Table 2). Mean differences ranged from + 2.4 to + 8.6 points (all *p* values < 0.001, Table 2). The pre-injury EQ-5D score of the total group of patients was 0.05 points higher compared to the Dutch population (*p* < 0.001, Table 2).

**Table 2:** Unadjusted differences in pre-injury scores and the Dutch population norms

	Pre-injury scores of injured patients			Dutch population [25, 26]			Diff.	*p* value
	*N*	Mean (SD)	95% CI	*N*	Mean (SD)	95% CI		
SMFA-NL								
Function Index	508	94.2 (9.5)	93.4–95.0	633	88.5 (13.1)	87.4–89.5	5.7	< 0.001
Bother Index	567	92.5 (13.3)	91.4–93.6	822	86.9 (17.5)	85.8–88.2	5.6	< 0.001
Upper extremity dysfunction	578	97.5 (8.4)	96.9–98.2	831	95.1 (11.8)	94.3–95.9	2.4	< 0.001
Lower extremity dysfunction	550	95.3 (11.1)	94.4–96.4	741	89.9 (14.1)	88.8–90.9	5.4	< 0.001
Problems with daily activities	515	93.4 (12.7)	92.3–94.5	706	87.8 (17.1)	89.5–89.1	5.6	< 0.001
Mental and emotional problems	568	87.3 (14.4)	86.1–88.4	831	78.7 (17.3)	77.6–79.9	8.6	< 0.001
EQ-5D	527	0.92 (0.17)	0.91–0.94	2667	0.87 (0.18)	0.86–0.88	0.05	< 0.001

Diff.: Unadjusted mean difference in score between the injured patient sample and the Dutch normative data samples

The adjusted mean differences between pre-injury scores of injured patients and the Dutch population ranged from + 0.8 to + 6.8 points (shown in Tables 3, 4). At the Bonferroni corrected alpha level, the adjusted differences between the injured patients and the Dutch population were significant for all subscales, except for *upper extremity dysfunction* subscale (+ 0.8 points [95% CI − 0.4 to 2.1], *p* = 0.2). For all subscales, the number of chronic health conditions was found to be the strongest confounders for the difference in health status between injured patients and the general population. Chronic health conditions reduced the estimate of the difference in score between injured patients and the Dutch population, ranging from a 32% reduction on the *mental and emotional problems* subscale to a 65% reduction on the *upper extremity dysfunction* subscale.

**Table 3:** Adjusted difference between injured patients and the Dutch population for the indices of the SMFA-NL

	Original indices					
	Function Index *N* = 898			Bother Index *N* = 1068		
	*B*	95% CI	*p* value	*B*	95% CI	*p* value
Injured versus Dutch population	3.6	2.2 to 4.9	< 0.001	3.0	1.4 to 4.7	< 0.001
Female	− 1.3	− 2.6 to 0.0	0.052	− 1.2	− 2.8 to 0.4	0.1
Age						
18–24	0			0		
25–34	− 1.2	− 3.4 to 1.1	0.3	− 2.8	− 5.6 to − 0.1	0.04
35–44	− 1.0	− 3.2 to 1.2	0.4	− 3.5	− 6.3 to − 0.7	0.02
45–54	− 1.0	− 3.3 to 1.3	0.4	− 3.1	− 6.0 to − 0.3	0.03
55–64	− 2.2	− 4.5 to 0.1	0.06	− 4.3	− 7.2 to − 1.4	0.003
65–75	0.8	− 1.8 to 3.4	0.053	1.3	− 1.8 to 4.4	0.4
Education						
Elementary school	0			0		
High school	2.9	− 1.1 to 6.8	0.2	1.0	− 3.5 to 5.5	0.7
College	3.2	− 0.8 to 7.1	0.1	2.0	− 2.5 to 6.6	0.4
Bachelor or higher	3.0	− 0.9 to 7.0	0.1	2.9	− 1.6 to 7.5	0.2
Chronic health conditions						
None	0			0		
One	− 4.5	− 6.0 to − 3.0	< 0.001	− 5.4	− 7.3 to − 3.5	< 0.001
Two	− 9.9	− 12.0 to − 7.8	< 0.001	− 12.5	− 14.9 to − 10.0	< 0.001
≥ Three	− 23.0	− 25.6 to − 20.4	< 0.001	− 26.8	− 29.6 to − 24.0	< 0.001
With partner	0.3	− 1.2 to 1.7	0.3	0.9	− 0.8 to 2.7	0.3
Intercept	95.9	91.8 to 99.9	< 0.001	97.9	93.1 to 102.7	< 0.001

B: Regression Coefficient. Results in bold reflect the adjusted difference in SMFA-NL pre-injury score compared to the Dutch population norm. For variables with multiple levels, the level designated as reference has a regression coefficient set to 0

**Table 4:** Adjusted difference between injured patients and the Dutch population for the subscales of the SMFA-NL

	Subscales of the SMFA-NL											
	Upper extremity dysfunction *N* = 1080			Lower extremity dysfunction *N* = 1003			Problems with daily activities *N* = 955			Mental and emotional Problems *N* = 1078		
	*B*	95% CI	*p* value	*B*	95% CI	*p* value	*B*	95% CI	*p* value	*B*	95% CI	*p* value
Injured versus Dutch population	0.8	− 0.4 to 2.1	0.2	3.7	2.3 to 5.0	< 0.001	2.8	1.1 to 4.6	0.001	6.8	5.1 to 8.6	< 0.001
Female	1.4	− 2.5 to − 0.2	0.2	− 0.5	− 1.8 to 0.8	0.4	− 1.5	− 3.1 to 0.2	0.08	− 2.5	− 4.2 to − 0.8	0.004
Age												
18–24	0			0			0			0		
25–34	− 0.8	− 2.8 to 1.2	0.4	0.0	− 2.3 to 2.4	1.0	− 2.8	− 5.6 to 0.0	0.054	− 2.0	− 4.9 to 0.9	0.2
35–44	− 1.4	− 3.4 to 0.6	0.2	− 0.7	− 3.0 to 1.6	0.5	− 2.8	− 5.6 to 0.1	0.06	− 1.6	− 4.5 to 1.4	0.3
45–54	− 0.6	− 2.7 to 1.4	0.6	− 0.4	− 2.8 to 1.9	0.7	− 3.2	− 6.1 to − 0.2	0.04	0.0	− 3.0 to 3.0	1.0
55–64	− 2.4	− 4.4 to − 0.3	0.03	− 1.8	− 4.2 to 0.6	0.1	− 4.8	− 7.8 to − 1.9	0.001	0.0	− 3.0 to 3.0	1.0
65–75	− 0.6	− 2.8 to 1.6	0.6	0.1	− 2.5 to 2.7	0.9	0.1	− 3.2 to 3.3	1.0	6.6	3.3 to 9.8	< 0.001
Education												
Elementary School	0			0			0			0		
High School	2.6	− 0.6 to 5.8	0.1	3.7	− 0.1 to 7.6	0.06	− 0.9	− 5.9 to 4.1	0.7	5.9	1.1 to 10.7	0.02
College	2.1	− 1.1 to 5.8	0.2	3.8	− 0.2 to 7.7	0.06	0.0	− 5.1 to 5.1	1.0	7.8	2.9 to 12.6	0.002
Bachelor or higher	1.7	− 1.5 to 5.0	0.3	4.4	0.5 to 8.3	0.03	0.2	− 4.9 to 5.3	0.9	7.7	2.8 to 12.5	0.002
Chronic health conditions												
None	0			0			0			0		
One	− 2.2	− 3.6 to − 0.8	0.002	− 3.8	− 5.3 to − 2.2	< 0.001	− 6.0	− 8.0 to − 4.1	< 0.001	− 5.6	− 7.6 to − 3.7	< 0.001
Two	− 3.6	− 5.9 to − 1.9	< 0.001	− 9.0	− 11.0 to − 6.9	< 0.001	− 13.2	− 15.8 to − 10.6	< 0.001	− 13.3	− 15.9 to − 10.7	< 0.001
≥ Three	− 14.6	− 16.6 to − 12.6	< 0.001	− 23.1	− 25.5 to − 20.6	< 0.001	− 27.2	− 30.3 to − 24.1	< 0.001	− 24.7	− 27.7 to − 21.8	< 0.001
With partner	0.7	− 0.6 to 2.0	0.3	− 0.2	− 1.7 to 1.3	0.8	0.2	− 1.6 to 2.1	0.8	2.8	0.9 to 4.7	0.004
Intercept	98.6	95.1 to 102.0	< 0.001	96.3	92.2 to 100.4	< 0.001	101.0	95.8 to 106.3	< 0.001	85.0	80.0 to 90.0	< 0.001

B: Regression Coefficient. Results in bold reflect the adjusted difference in SMFA-NL pre-injury score compared to the Dutch population norm. For variables with multiple levels, the level designated as reference has a regression coefficient set to 0

### Sensitivity analysis

In part one of the sensitivity analysis, injured patients had a significantly higher likelihood of scoring the maximum SMFA-NL score, on all indices and subscales (Appendix Tables 5, 6, 7), compared to the Dutch population. Odds ratios ranged from 1.95 [95% CI 1.2–4.4], *p* < 0.001 on the *bother index*, to 3.96 [95% CI 2.92–5.37], *p* < 0.001 on the *lower extremity dysfunction* subscale. For all subscales, chronic health conditions significantly decreased the probability of scoring the maximum SMFA score (Appendix Tables 5, 6, 7).

In part two of the sensitivity analysis (only patients with a sub-maximal score), injured patients showed a significantly better score on the *function index* (2.8 points [95% CI 1.2–4.4], *p* < 0.001, Appendix Table 5) and the *mental and emotional problems* subscale (4.9 points, [95% CI 2.9–6.9], *p* < 0.001, Appendix Table 7), compared to the Dutch population. The difference in score between the injured patients and the Dutch population was not significantly different for the *bother index, upper extremity dysfunction, lower extremity dysfunction*, and *problems with daily activities* subscales (Appendix Tables 5, 6, 7). For all subscales, the presence of chronic health conditions was significantly associated with reporting a lower score (Appendix Tables 5, 6, 7).

## Discussion

The present study showed that injured patients reported significantly better pre-injury scores compared to the Dutch population for both the condition-specific SMFA-NL and the generic EQ-5D questionnaires. Adjustment for general characteristics resulted in a reduction of the differences between pre-injury health status of injured patients and the Dutch population, yet it remained significantly different. The reduction of this difference in health status between both samples was mainly due to the lower number of chronic health conditions reported by injured patients.

It is important to evaluate whether the differences in health status are clinically relevant. To the best of our knowledge, there is no known minimally important difference (MID) value of the SMFA [31]. Hence, there is no clear reference available that can be used to indicate which difference between groups may be considered clinically relevant. However, the differences were smaller than the standard error of measurement of the SMFA-NL, which ranged from 7.8 points for the function index, to 11.3 points for the mental and emotional problems subscale [16]. We think that the adjusted differences in health status between the injured patients and the Dutch population were too small to reflect a clinically relevant difference. This was supported by part two of the sensitivity analysis, which showed that among patients with a sub-maximal score, there was no evidence of a difference in health status between injured patients and the Dutch population for four of the six scales.

Though there was little evidence of a difference in health status between the injured patients and the Dutch population, among patients with a sub-maximal score (part two of the sensitivity analysis), this conclusion may not be directly translated to patients that reached the limit of the scale (i.e., a score of 100 points). The sensitivity analysis (part one) showed that injured patients were significantly more likely to reach the maximal score than the Dutch population. The increased likelihood of reaching the maximal score may indicate that there could be a difference in health status between the injured patients and the Dutch population ‘above’ the maximal SMFA-NL score of 100 points. However, since 100 points was the upper limit of the scale, the difference in health status between both groups could not be further quantified. This was a limitation of this study and may be subject of further research using a questionnaire that is less susceptible to ceiling effects.

Regarding the EQ-5D, one MID value of 0.08 points has been reported to compare groups of patients with musculoskeletal conditions [32, 33]. This value was not reported in an injury-specific study population, but was calculated from a sample of patients undergoing total hip arthroplasty. Based on this MID, the difference between injured patients and the normative data of the EQ-5D found in our study (an unadjusted difference of 0.05 points) was perceived as being not a clinically important difference. In addition, the EQ-5D score difference was not adjusted for patient characteristics and may be smaller after adjustment for patient characteristics.

The unadjusted differences found in the present study are in line with previous research on generic HRQoL instruments. In a systematic review, Scholten et al. concluded that recalled pre-injury health status consistently exceeded population norms in patients with traumatic injuries [34]. In a sample of patients with a broad range of traumatic injuries, Watson et al. used the SF-36 and reported higher pre-injury scores on both the physical and mental domains [12]. The differences found in the study of Watson et al. were of a similar magnitude to the unadjusted differences found in the present study. Wilson et al. used the EQ-5D in a large sample of 2842 patients that sustained various traumatic injuries, and reported that pre-injury health status was 0.12 points higher than the health status of the general population [8].

In several previous studies, it has been discussed that the (unadjusted) difference between injured patients and the general population may be explained in terms of recall bias or response shift [8, 9, 12, 34]. In this context, response shift means that the experience of poorer health status after the injury may have inflated the patient’s valuation of recalled pre-injury health status [34, 35]. Alternatively, it was hypothesized that injured patients may be a specific sub-sample of the general population [8, 9, 12, 34]. However, in these studies, the differences were never adjusted for patient characteristics. The present study showed that controlling for patient characteristics led to a reduction of the difference in pre-injury health status and health status of the general population. Having one or more chronic health conditions was of greater influence on the difference in health status, than originating either from the group of injured patients or the Dutch population. Hence, though the present study was not able to quantify response shift or recall bias, the findings imply that the differences between recalled pre-injury health status and general population norms may for an important part be explained by differences in general characteristics and in particular the number of chronic health conditions.

Prospective evaluation of pre-injury health status is preferred, since it is not subject to bias and response shift due to sustaining the injury [34]. However, in clinical practice, prospective evaluation is generally not feasible. The use of normative data has been advocated, since it provides pre-injury estimates that are free of recall bias and response shift [34]. In addition, the use of normative data relieves administrative burden on patients. However, the use of normative data relies on the assumption that the population norms are an accurate reflection of injured patients. The (adjusted) difference in health status between patients with a broad range of traumatic injuries and the general population norms is small [34]. However, this may not be applicable to all injured patients. In specific samples, such as hip fractures, patients have a worse pre-injury health status opposed to the general population [36, 37]. In contrast, patients with gun-shot injuries and traumatic brain injury report a high pre-injury health status [38, 39]. It has been suggested that patients with specific injuries are likely to respectively have a poorer or better general health than the general population in terms of socioeconomic status or comorbidities [34]. Due to the underlying assumptions for the use of normative data, the representativeness of the normative data for the study sample should be considered carefully before being used, especially in patients with specific injuries. If population norms are used as a proxy for pre-injury health status, they should be adjusted for differences in general characteristics.

Recalled pre-injury scores on the other hand are also subject to debate. As outlined earlier, there is a susceptibility to two biasing factors. Firstly, patients may have remembered their pre-injury health state incorrectly, thereby inducing recall bias. Recall bias may lead to an overestimation of patients their pre-injury health status [40, 41]. However, when patients recall their pre-injury health status shortly after the injury, recall bias may be limited. A two-week interval is generally considered appropriate to limit recall bias [28, 42]. Secondly, response shift may operate. Since patients evaluate their pre-injury health status after the injury, the injury itself may have changed patients’ perception of their pre-injury health status, due to a change in internal valuation of what health is [35]. This may inflate the recalled pre-injury health status. In the absence of prospectively assessed pre-injury health status, it is not possible to quantify response shift. Nonetheless, others have argued that post-injury assessment of pre-injury health status may have its advantages. It enables patients to value their pre-injury health status based on newly learned information that could not have been gained before the injury and is not present in population norms [34, 43]. In addition, recalled pre-injury health status enables that pre- and post-injury health status evaluation can be based on the same set of internal values, which has been suggested to be preferable in terms of validity and reliability [34, 43, 44].

## Limitations of the present study

One of the limitations of this study was that the two PROMs that were used were susceptible to detecting ceiling effects. This is a known limitation of both the SMFA-NL and EQ-5D [14, 16, 45]. Because pre-injury and general population health status were considered relatively ‘healthy’ conditions, ceiling effects were expected. A sensitivity analysis by means of a two-part model was used to account for the ceiling effects on the SMFA-NL. The sensitivity analysis could not be performed for the EQ-5D since the original dataset could not be obtained.

Additional differences between injured patients and the general population may be explained by other variables, such as socioeconomic status, and additional chronic health conditions such as kidney disease, levels of pre-injury physical activity, and mental health [34]. However, these variables were not available in this study.

The sample size of the study was considered adequate and the response rate of 43% was considered reasonable for an injured patient population, however, it may have introduced selection bias [46].

The differences in the applied methods of administration of the SMFA-NL and EQ-5D might be considered a limitation. The injured patients completed the questionnaires on paper, while the normative data of the SMFA-NL were administered electronically [25]. The EQ-5D normative data were mainly sampled using internet web forms [26]. In a meta-analysis, it was concluded that there is extensive evidence of the equivalence of on-paper and electronically administered PROMs [47]. We believe that the mode of administration had no influence on the differences between the study samples.

To obtain pre-injury health status, patients were asked to report their health status of the week before their injury. The recall period both PROMs was slightly changed from the original PROM. This was considered a limitation of this study, since it is preferable to completely re-evaluate the validity and reliability of a PROM when any change is made to it [48, 49]. Though no standard recall period exists, typically shorter recall periods are preferred, and must be based on the purpose of the assessment [50]. The recall interval of the adjusted question was considered was very similar to the original question, appropriate for both measures and short enough such that the effects on the validity, reliability, and recall bias of both questionnaires would be limited.

In future studies where pre-injury data are not available, adjusted normative data may be used to compare groups of patients that sustained general trauma. Prospective (population-wide) studies may provide insight in the effects of recall bias and response shift on pre-injury health status.

## Conclusion

This study provided insight into differences in population characteristics and pre-injury health status of injured patients, compared to the Dutch general population. For both the generic HRQoL and condition-specific measures, injured patients reported a better pre-injury health status than the general population. However, general characteristics explained an important part of the difference in health status between injured patients and the general population. Within the detectable range of the scale, adjusted differences between the recalled pre-injury health status of injured patients and the general population were considered not clinically relevant.

## Appendix

See Tables 5, 6 and 7.

**Table 5:** Two-part model for the Function Index and Bother Index

	Function Index						Bother Index					
	Part one *N* = 898			Part two *N* = 760			Part one *N* = 1068			Part two *N* = 661		
	OR	95%CI	*p* value	*B*	95% CI	*p* value	OR	95% CI	*p* value	B	95% CI	*p* value
Injured versus Dutch population	3.85	2.52 to 5.87	< 0.001	2.8	1.2 to 4.4	< 0.001	1.95	1.47 to 2.59	< 0.001	1.6	− 0.9 to 4.2	0.2
Female	0.61	0.40 to 0.92	0.02	− 1.0	− 2.4 to 0.5	0.2	0.88	0.67 to 1.17	0.4	− 1.5	− 3.8 to 0.8	0.2
Age												
18–24	1			0			1			0		
25–34	0.89	0.46 to 1.72	0.7	− 1.4	− 4.0 to 1.1	0.3	0.91	0.57 to 1.43	0.7	− 4.9	− 9.1 to − 0.6	0.02
35–44	1.32	0.68 to 2.57	0.4	− 1.4	− 4.0 to 1.2	0.3	1.09	0.68 to 1.75	0.7	− 6.3	− 10.6 to − 2.0	0.004
45–54	1.20	0.60 to 2.38	0.6	− 1.5	− 4.1 to 0.1	0.3	0.80	0.49 to 1.28	0.3	− 4.3	− 8.4 to − 0.2	0.04
55–64	0.68	0.31 to 1.47	0.3	− 2.1	− 4.7 to 0.4	0.1	0.41	0.24 to 0.69	< 0.001	− 3.6	− 7.6 to 0.5	0.08
65–75	0.78	0.29 to 2.07	0.6	1.0	− 1.9 to 3.9	0.5	0.71	0.41 to 1.25	0.2	1.8	− 2.5 to 6.2	0.4
Education												
Elementary school	1			0			1			0		
High school	1.68	0.35 to 7.95	0.5	2.7	− 1.6 to 7.0	0.2	1.87	0.73 to 4.76	0.2	− 0.3	− 6.1 to 5.4	0.9
College	3.42	0.72 to 16.15	0.1	2.6	− 1.8 to 7.1	0.2	2.39	0.93 to 6.13	0.07	0.4	− 5.5 to 6.3	0.9
Bachelor or higher	2.39	0.51 to 11.29	0.3	2.6	− 1.8 to 7.0	0.2	2.41	0.95 to 6.15	0.07	2.2	− 3.6 to 8.1	0.4
Chronic health conditions												
None	1			0			1			0		
One	0.45	0.29 to 0.71	< 0.001	− 4.6	− 6.3 to − 2.8	< 0.001	0.54	0.40 to 0.74	< 0.001	− 6.7	− 9.5 to − 3.8	< 0.001
Two	0.07	0.02 to 0.30	< 0.001	− 9.3	− 11.6 to − 7.1	< 0.001	0.23	0.14 to 0.36	< 0.001	− 12.4	− 15.7 to − 9.1	< 0.001
≥ Three	0.08	0.01 to 0.58	0.01	− 22.6	− 25.4 to − 19.8	< 0.001	0.07	0.03 to 0.16	< 0.001	− 25.3	− 28.9 to − 21.8	< 0.001
With partner	1.11	0.70 to 1.77	0.7	0.2	− 1.5 to 1.9	0.8	0.96	0.70 to 1.31	0.8	2.0	− 0.5 to 4.6	0.1
Intercept	0.07	0.01 to 0.34	< 0.001	92.1	87.3 to 96.8	< 0.001	− 0.67	0.19 to 1.37	0.2	92.8	86.1 to 99.5	< 0.001

Part One: part one of the two-part model (logistic regression, entire sample). Part two: part two of the two-part model (linear regression, only patients with sub-maximal score)

In bold: results of the comparison of the injured patients and the Dutch population. For variables with multiple levels, the level designated as reference has a regression coefficient set to 0 and an odds ratio set to 1

*OR* odds ratio, *95% CI* 95% confidence interval, *B* regression coefficient

**Table 6:** Two-part model for upper extremity dysfunction and lower extremity dysfunction subscales

	Upper extremity dysfunction						Lower extremity dysfunction					
	Part one *N* = 1080			Part two *N* = 237			Part one *N* = 1003			Part two *N* = 550		
	OR	95% CI	*p* value	B	95% CI	*p* value	OR	95% CI	*p* value	B	95% CI	*p* value
Injured versus Dutch population	2.33	1.58 to 3.44	< 0.001	− 4.4	− 9.6 to 0.8	0.09	3.96	2.92 to 5.37	< 0.001	2.2	− 0.4 to 4.7	0.09
Female	0.53	0.37 to 0.74	< 0.001	− 2.6	− 6.7 to 1.6	0.2	0.98	0.73 to 1.31	0.9	− 1.2	− 3.4 to 1.0	0.3
Age												
18–24	1			0			1			0		
25–34	1.05	0.54 to 2.07	0.9	− 9.4	− 18.6 to − 0.1	0.047	1.27	0.78 to 2.09	0.3	− 1.8	− 5.8 to 2.3	0.4
35–44	0.70	0.37 to 1.32	0.3	− 6.0	− 14.6 to 2.7	0.6	1.23	0.75 to 2.03	0.4	− 2.8	− 6.8 to 1.2	0.2
45–54	0.64	0.34 to 1.20	0.2	− 2.3	− 10.8 to 6.3	0.9	0.90	0.54 to 1.48	0.7	− 1.2	− 5.1 to 2.7	0.5
55–64	0.26	0.14 to 0.47	< 0.001	− 0.5	− 8.1 to 7.1	0.5	0.53	0.31 to 0.90	0.02	− 1.5	− 5.3 to 2.4	0.4
65–75	0.58	0.30 to 1.10	0.1	− 2.8	− 10.9 to 5.4	0.7	0.68	0.37 to 1.23	0.2	− 0.2	− 4.3 to 3.9	0.9
Education												
Elementary school	1			0			1			0		
High school	2.84	1.28 to 6.33	0.01	1.3	− 6.8 to 9.5	0.7	1.88	0.76 to 4.65	0.2	4.9	− 0.8 to 10.5	0.09
College	3.21	1.40 to 7.37	0.006	− 2.5	− 11.2 to 6.3	0.6	2.42	0.97 to 6.05	0.06	4.1	− 1.7 to 9.8	0.2
Bachelor or higher	2.23	0.99 to 5.02	0.052	− 1.3	− 9.7 to 7.1	0.8	2.47	1.00 to 6.11	0.051	5.3	− 0.5 to 11.0	0.07
Chronic health conditions												
None	1			0			1			0		
One	0.50	0.33 to 0.76	0.001	− 7.3	− 13.3 to 1.3	0.02	0.48	0.35 to 0.67	< 0.001	− 5.2	− 7.8 to − 2.5	< 0.001
Two	0.32	0.20 to 0.51	< 0.001	− 7.4	− 13.6 to 1.1	0.02	0.22	0.13 to 0.35	< 0.001	− 9.2	− 12.2 to − 6.1	< 0.001
≥ Three	0.09	0.05 to 0.15	< 0.001	− 18.3	− 24.2 to 12.5	< 0.001	0.08	0.04 to 0.18	< 0.001	− 22.5	− 25.8 to − 19.1	< 0.001
With partner	1.19	0.82 to 1.73	0.4	1.2	− 3.4 to 5.8	0.6	0.80	0.58 to 1.11	0.2	0.3	− 2.2 to 2.8	0.8
Intercept	4.73	1.79− 12.49	0.001	97.3	86.0 to 108.5	< 0.001	0.49	0.18 to 1.30	0.2	89.9	83.4 to 96.4	< 0.001

Part One: part one of the two-part model (logistic regression, entire sample). Part two: part two of the two-part model (linear regression, only patients with sub-maximal score)

In bold: results of the comparison of the injured patients and the Dutch population. For variables with multiple levels, the level designated as reference has a regression coefficient set to 0 and an odds ratio set to 1

*OR* odds ratio, *95% CI* 95% confidence interval, *B* regression coefficient

**Table 7:** Two-part model for the problems with daily activities and mental and emotional problems subscales

	Problems with daily activities						Mental and emotional problems					
	Part one *N* = 955			Part two *N* = 647			Part one *N* = 1078			Part TWO *N* = 900		
	OR	95% CI	*p* value	*B*	95% CI	*p* value	OR	95% CI	*p* value	*B*	95% CI	*p* value
Injured versus Dutch population	2.95	2.16 to 4.03	< 0.001	1.0	− 1.5 to 3.5	0.4	3.49	2.42 to 5.04	< 0.001	4.9	2.9 to 6.9	< 0.001
Female	0.75	0.54 to 1.01	0.06	− 1.4	− 3.7 to 0.9	0.2	0.72	0.50 to 1.03	0.07	− 2.0	− 3.9 to − 0.2	0.03
Age												
18–24	1			0			1			0		
25–34	0.91	0.56 to 1.50	0.7	− 4.8	− 9.0 to − 0.7	0.02	1.18	0.64 to 2.16	0.6	− 2.9	− 6.1 to 0.2	0.07
35–44	0.94	0.57 to 1.56	0.8	− 4.7	− 8.9 to − 0.6	0.02	1.72	0.93 to 3.19	0.08	− 3.2	− 6.5 to 0.1	0.06
45–54	0.85	0.51 to 1.43	0.5	− 4.8	− 8.9 to − 0.7	0.02	1.35	0.72 to 2.54	0.4	− 0.6	− 3.8 to 2.6	0.07
55–64	0.35	0.19 to 0.63	< 0.001	− 4.5	− 8.5 to − 0.5	0.02	0.98	0.49 to 1.98	1.0	0.1	− 3.1 to 3.3	1.0
65–75	0.49	0.25 to 0.96	0.04	0.4	− 3.9 to 4.0	0.8	1.44	0.67 to 3.11	0.4	6.4	2.9 to 9.8	< 0.001
Education												
Elementary school	1			0			1			0		
High school	1.05	0.38 to 2.90	0.9	− 1.1	− 7.6 to 5.5	0.8	3.13	0.69 to 14.2	0.1	4.5	− 0.4 to 9.5	0.07
College	1.49	0.53 to 4.16	0.4	− 0.7	− 7.4 to 6.0	0.8	4.81	1.05 to 21.9	0.04	6.0	0.9 to 11.0	0.02
Bachelor or higher	1.43	0.52 to 3.98	0.5	− 0.4	− 7.0 to 6.3	0.9	2.86	0.63 to 13.0	0.2	6.9	2.0 to 11.9	0.006
Chronic health conditions												
None	1			0			1			0		
One	0.54	0.38 to 0.75	< 0.001	− 7.6	− 10.4 to − 4.9	< 0.001	0.41	0.27 to 0.62	< 0.001	− 4.4	− 6.6 to − 2.2	< 0.001
Two	0.17	0.09 to 0.32	< 0.001	− 13.2	− 16.5 to − 9.9	< 0.001	0.10	0.04 to 0.25	< 0.001	− 10.9	− 13.5 to − 8.2	< 0.001
≥ Three	0.10	0.03 to 0.28	< 0.001	− 27.4	− 31.2 to − 23.5	< 0.001	0.10	0.03 to 0.32	< 0.001	− 22.5	− 25.6 to − 19.5	< 0.001
With partner	0.93	0.66 to 1.31	0.7	0.7	− 1.8 to 3.3	0.6	1.24	0.82 to 1.88	0.3	2.9	0.9 to 5.0	0.004
Intercept	0.57	0.19 to 1.69	0.3	97.6	90.3 to 104.9	< 0.001	0.04	0.01 to 0.22	< 0.001	76.8	72.3 to 82.4	< 0.001

Part One: part one of the two-part model (logistic regression, entire sample). Part two: part two of the two-part model (linear regression, only patients with sub-maximal score)

In bold: results of the comparison of the injured patients and the Dutch population. For variables with multiple levels, the level designated as reference has a regression coefficient set to 0 and an odds ratio set to 1

*OR* odds ratio, *95% CI* 95% confidence interval, *B* regression coefficient
